# Comparison of Anti-Oxidant and Anti-Inflammatory Effects between Fresh and Aged Black Garlic Extracts

**DOI:** 10.3390/molecules21040430

**Published:** 2016-03-30

**Authors:** Yi Yeong Jeong, Ji Hyeon Ryu, Jung-Hye Shin, Min Jung Kang, Jae Ran Kang, Jaehee Han, Dawon Kang

**Affiliations:** 1 Departments of Allergy and Respiratory Medicine, Gyeongsang National University and Gyeongsang National University Hospital, Jinju 660-751, Korea; dr202202@naver.com; 2 Departments of Physiology, College of Medicine, Gyeongsang National University, Jinju 660-751, Korea; wlgus9217@naver.com (J.H.R.); jheehan@gnu.ac.kr (J.H.); 3 Department of Research and Development, Namhae Garlic Research Institute, Namhae 668-812, Korea; whanbee@hanmail.net (J.-H.S.); jung-75@hanmail.net (M.J.K.); rani921@hanmail.net (J.R.K.); 4 Institute of Health Sciences, Gyeongsang National University, Jinju 660-751, Korea

**Keywords:** anti-inflammatory agents, antioxidants, garlic, NF-kappa B, sugar

## Abstract

Numerous studies have demonstrated that aged black garlic (ABG) has strong anti-oxidant activity. Little is known however regarding the anti-inflammatory activity of ABG. This study was performed to identify and compare the anti-oxidant and anti-inflammatory effects of ABG extract (ABGE) with those of fresh raw garlic (FRG) extract (FRGE). In addition, we investigated which components are responsible for the observed effects. Hydrogen peroxide (H_2_O_2_) and lipopolysaccharide (LPS) were used as a pro-oxidant and pro-inflammatory stressor, respectively. ABGE showed high ABTS and DPPH radical scavenging activities and low ROS generation in RAW264.7 cells compared with FRGE. However, inhibition of cyclooxygenase-2 and 5-lipooxygenase activities by FRGE was stronger than that by ABGE. FRGE reduced PGE_2_, NO, IL-6, IL-1β, LTD_4_, and LTE_4_ production in LPS-activated RAW264.7 cells more than did ABGE. The combination of FRGE and sugar (galactose, glucose, fructose, or sucrose), which is more abundant in ABGE than in FRGE, decreased the anti-inflammatory activity compared with FRGE. FRGE-induced inhibition of NF-κB activation and pro-inflammatory gene expression was blocked by combination with sugars. The lower anti-inflammatory activity in ABGE than FRGE could result from the presence of sugars. Our results suggest that ABGE might be helpful for the treatment of diseases mediated predominantly by ROS.

## 1. Introduction

It has been suggested that the production of excessive reactive oxygen species (ROS), which causes oxidative stress [1] and protein oxidation [2], is involved in the pathogenesis of many types of inflammatory disease, including atherosclerosis, rheumatoid arthritis, cancer, and allergies. Numerous studies have demonstrated that high levels of ROS induce inflammation [3,4,5] and that such inflammation complicates diseases. It has been believed that anti-oxidants exert anti-inflammatory effects; therefore, phytochemicals with anti-oxidant effects have been developed to treat inflammatory disease.

Garlic (*Allium sativum*), which contains a variety of phytochemicals, has been known as a medicinal food with anti-oxidant and anti-inflammatory activities [6,7,8,9]. In particular, aged black garlic (ABG), an aged garlic formulation containing high levels of organosulfur compounds, has strong anti-oxidant effects compared with fresh raw garlic (FRG) [10,11]. ABG is a functional food and is primarily marketed in Asia; it is produced through aging procedures at high temperature and humidity over a long time [10,12]. During the aging processes, the levels of compounds with anti-oxidant activity, such as pyruvate and *S*-allylcysteine (SAC), are increased [12,13,14,15,16]. Aged garlic extracts (AGE) including ABG contain water-soluble and lipid-soluble organosulfur compounds, but not allicin [17,18]. SAC and *S*-allylmercaptocysteine are the major unique water-soluble organosulfur compounds in AGE. Diallyl sulfide (DAS), diallyl disulfide (DADS), diallyl trisulfide (DATS), and diallyl tetrasulfide are lipid-soluble compounds in AGE. These organosulfur compounds derived from allicin contribute to the anti-oxidant activity of AGE [17,18]. Allicin present in crushed FRG, which is chemically unstable, is responsible for the anti-inflammatory effect of FRG [8,19]. These anti-oxidant activities of ABG may also lead to an anti-inflammatory action. However, few studies have considered the anti-inflammatory effects of ABG. Chloroform extract of ABG attenuates TNF-α-induced NF-κB activation in human umbilical vein endothelial cells [20]. In addition, methanol extract of ABG inhibits production of cyclooxygenase-2 (COX-2) and prostaglandin E_2_ (PGE_2_) by phorbol 12-myristate-13-acetate through inactivation of NF-κB [21].

The heating processes used to prepare ABG increase the concentration of sugars as well as of compounds with anti-oxidant activity [16,22,23]. Other than their sweet flavor, little is known regarding the anti-oxidant and/or anti-inflammatory effects of the sugars in ABG. Recent studies have reported that sugars induce inflammation [24,25,26] by increasing levels of pro-inflammatory cytokines and NF-κB activation [27,28,29].

This study was performed to compare the anti-oxidant and anti-inflammatory effects of ABG extract (ABGE) with FRG extract (FRGE). In addition, we investigated the effects of pyruvate and sugars on hydrogen peroxide (H_2_O_2_)- or lipopolysaccharide (LPS)-stimulated RAW264.7 cells. Furthermore, we analyzed NF-κB activation and the expression of pro-inflammatory genes to identify the mechanism underlying the anti-oxidant and anti-inflammatory effects of garlic extracts.

## 2. Results

### 2.1. High Anti-Oxidant Activity in ABGE

**Figure 1:**
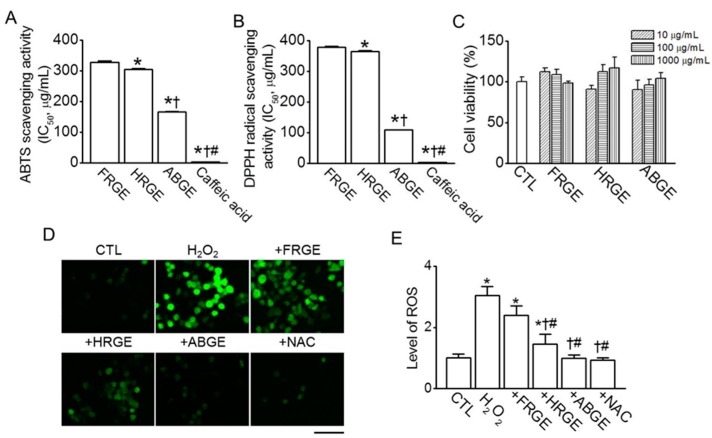
Antioxidant activity of garlic extracts. (**A**) ABTS and (**B**) DPPH radical scavenging activities of garlic extracts. Each bar represents the half maximal inhibitory concentration (IC_50_). * *p* < 0.05 compared with the FRGE treatment. ^†^ *p* < 0.05 compared with the HRGE treatment. ^#^ *p* < 0.05 compared with the ABGE treatment; (**C**) Effect of garlic extracts on viability of RAW264.7 cells. Different concentrations of garlic extracts were treated to cells for 24 h; (**D**,**E**) Effect of garlic extracts on H_2_O_2_-induced ROS generation in RAW264.7 cells. Cells were treated with 100 μM H_2_O_2_, 1000 μg/mL garlic extracts, or 3 mM NAC and then stained with H_2_DCFDA to evaluate ROS generation. The ROS levels in the cells were quantified using fluorescence microscopy after 3 h of treatment with the extract. The plus sign (+) represents conditions co-treated with H_2_O_2_. The scale bar represents 50 μm. * *p* < 0.05 compared with the control (CTL). ^†^ *p* < 0.05 compared with the H_2_O_2_ treatment. ^#^ *p* < 0.05 compared with the FRGE treatment.

It is well-known that garlic displays anti-oxidant properties. To compare the anti-oxidant activity among different types of garlic extract, ABTS and DPPH radical scavenging assays were performed. Among the garlic extracts, ABGE showed the highest ABTS and DPPH scavenging activity. The concentrations that induced 50% inhibition (IC_50_) of ABTS were 327.5 ± 5.6, 305.5 ± 1.9, and 166.3 ± 1.3 μg/mL in FRGE, heated raw garlic extract (HRGE), and ABGE, respectively (*n* = 5, Figure 1A). The IC_50_ of DPPH was 378.3 ± 3.4, 364.4 ± 3.2, and 108.1 ± 0.9 μg/mL in FRGE, HRGE, and ABGE, respectively (*n* = 5, Figure 1B). Caffeic acid, a phenolic compound found in coffee beans, was used as a positive control for ABTS and DPPH scavenging (~3.2 μg/mL). Caffeic acid has been known as an effective anti-oxidant in *in vitro* and *in vivo* anti-oxidant assays [30,31]. To determine the optimal concentration of garlic extracts for H_2_O_2_- or LPS-stimulated cellular response, RAW264.7 cells were incubated with different concentrations of garlic extracts. As shown in Figure 1C, different type of garlic extracts affects cell viability in a different manner. FRGE and ABGE showed similar cell viability at the concentration of 1000 μg/mL. Half of the RAW264.7 cell population was killed in the presence of 100 μM H_2_O_2_ for 24 h. The concentrations of other chemicals used in this study were also determined based on the results of an MTT assay. The anti-oxidant activity of garlic extracts was analyzed in the RAW264.7 cells treated with H_2_O_2_. As shown in Figure 1D, H_2_O_2_ markedly increased ROS levels in the RAW264.7 cells compared with the control. Pretreating with 1 mM N-acetyl-l-cysteine (NAC), which is a ROS scavenger, significantly reduced H_2_O_2_-induced ROS generation (*p* < 0.05). Similar to NAC, HRGE and ABGE, but not FRGE, attenuated the H_2_O_2_-induced ROS levels. Among the garlic extracts, ABGE showed the greatest ROS scavenging activity (Figure 1D,E).

### 2.2. Low Anti-Inflammatory Activity in ABGE

**Figure 2:**
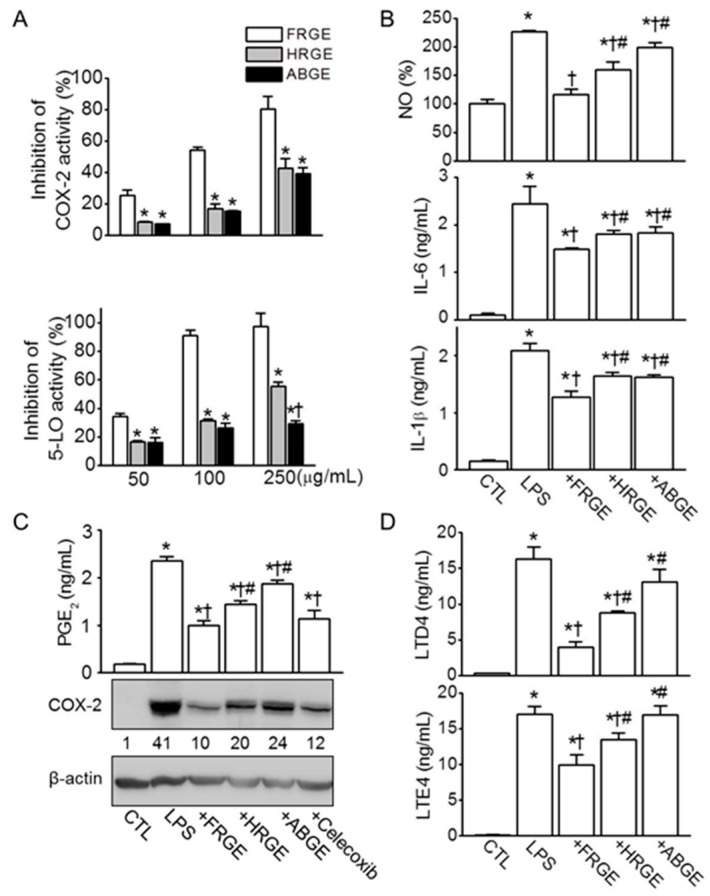
Anti-inflammatory activity of garlic extracts. (**A**) Inhibition of COX-2 and 5-LO activities by garlic extracts. The activities of garlic extracts were measured by calculating absorbance. * *p* < 0.05 compared with the FRGE treatment. ^†^ *p* < 0.05 compared with the HRGE treatment; (**B**) Inhibitory effects of garlic extracts on the production of NO and the release of pro-inflammatory cytokines (IL-6 and IL-1β) in LPS-activated RAW264.7 cells. The cells were pretreated for 2 h with garlic extract (1000 μg/mL) before stimulation with LPS (1 μg/mL) for 24 h; (**C**,**D**) Inhibitory effect of garlic extracts on the LPS-induced PGE_2_ secretion and COX-2 expression (**C**) and on the release of LTD4 and LTE4 (**D**). The concentrations of nitrite, IL-6, IL-1β, PGE2, LTD4, and LTE4 were measured in the collected media. Total protein isolated from the cells was subjected to western blot analysis for COX-2. The numbers below the blot represent the normalized ratio of the expression levels of COX-2 to those of β-actin for each lane. Equal quantities (30 μg) of total protein were loaded in each lane. β-actin was used as a loading control. * *p* < 0.05 compared with the control (CTL). ^†^ *p* < 0.05 compared with the LPS treatment. ^#^ *p* < 0.05 compared with the FRGE treatment.

To determine whether ABGE has anti-inflammatory activity, the ability of garlic extracts to inhibit COX-2 and 5-LO was analyzed. In addition, the anti-inflammatory activity was evaluated in LPS-activated RAW264.7 cells. In enzyme solutions, COX-2 and 5-LO activities decreased upon adding garlic extract in a dose-dependent manner (Figure 2A). Among the garlic extracts, FRGE showed the greatest inhibition of COX-2 and 5-LO activities (80.5% ± 7.8% and 97.4% ± 9.5% in FRGE *versus* 39.1% ± 3.8% and 29.5% ± 2.1% in ABGE at 250 μg/mL). HRGE and ABGE did not exhibit significant differences in COX-2 inhibition (*p* > 0.05), but ABGE showed less inhibition of 5-LO activity than did HRGE at 250 μg/mL (*p* < 0.05, Figure 2A). Epigallocatechin gallate (EGCG) used as a positive control inhibited COX-2 activity in an enzyme solution at a low concentration (80% reduction at 10 μg/mL). In LPS-activated RAW264.7 cells, FRGE markedly decreased the concentration of NO, IL-1β, and IL-6. This effect differed significantly from those of HRGE and ABGE (*p* < 0.05), and there was no significant difference between HRGE and ABGE (Figure 2B).

The changes in the concentrations of PGE_2_ and leukotriene (LTD4 and LTE4) and in the expression of COX-2 induced by the garlic extract were measured in LPS-activated RAW264.7 cells. LPS increased PGE_2_ production and COX-2 expression (Figure 2C). Pretreatment with garlic extract decreased the LPS-induced increases in PGE_2_ production and COX-2 expression. As shown in Figure 2C, the inhibitory effect was greatest in the FRGE treatment, and the effect of FRGE was similar to that of celecoxib, a COX-2 inhibitor. The effects of HRGE and ABGE on the decrease in PGE_2_ and COX-2 in the LPS-activated RAW264.7 cells were significantly smaller than that of FRGE treatment (*p* < 0.05). LPS also increased the production of LTD4 and LTE4 in the RAW264.7 cells. FRGE and HRGE, but not ABGE, significantly lowered the production of LTD4 and LTE4 in LPS-activated RAW264.7 cells (*p* < 0.05). The production of LTD4 was decreased by 76%, 46%, and 20% by FRGE, HRGE, and ABGE, respectively. The production of LTE4 was decreased by 42% in the FRGE treatment, whereas ABGE did not notably inhibit LTE4 production (Figure 2D).

### 2.3. Pyruvate is Responsible for the High Anti-Oxidant Effects of ABGE

To identify whether and which components in ABGE underlie its high anti-oxidant and low anti-inflammatory effects compared with FRGE, we investigated the effects of allicin, pyruvate, and sugars on the production of ROS, NO, and PGE_2_. We first measured the chemical concentration in garlic extracts. As shown in Table 1, the concentrations of pyruvate, thiosulfinate, sugars, and SAC in ABGE were higher than those in FRGE and HRGE. Allicin was not detected in ABGE. Pyruvate, which is abundant in ABGE, significantly decreased ROS generation (*p* < 0.05), similar to ABGE, but pyruvate had a weaker inhibitory effect on H_2_O_2_-induced ROS generation than did ABGE. However, when combined with FRGE, which did not show a significant decrease in H_2_O_2_-induced ROS generation, pyruvate significantly decreased its anti-oxidant effect. The rate of ROS reduction by the combination of FRGE and pyruvate was between the rates for FRGE and pyruvate, indicating that pyruvate has anti-oxidant activity, but that some components of FRGE inhibit the anti-oxidant activity of pyruvate.

**Figure 3:**
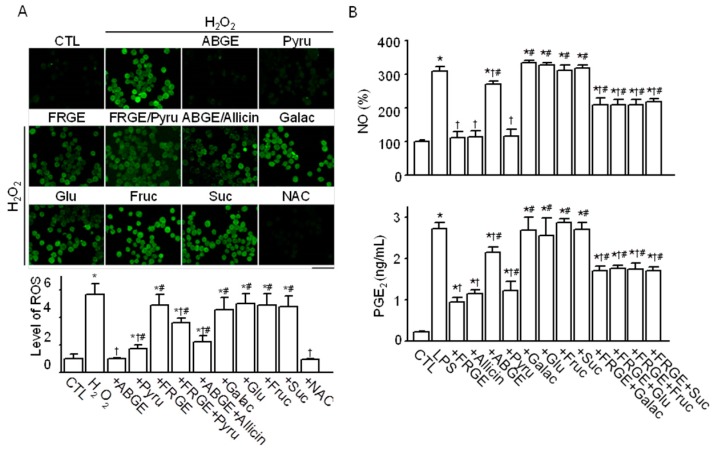
High anti-oxidant and low anti-inflammatory effects of ABGE compared with FRGE. (**A**) High anti-oxidant effect of ABGE. Pyruvate, not sugar, is responsible for the anti-oxidant effect of ABGE. RAW264.7 cells were incubated with H_2_DCFDA to evaluate ROS generation. The ROS levels were quantified using fluorescence microscopy after 3 h of treatment with 100 μM pyruvate, 1 μM allicin, 100 μM sugars, 1000 μg/mL garlic extracts, or 1 mM NAC in the H_2_O_2_ (100 μM)-treated cells. The scale bar represents 50 μm. * *p* < 0.05 compared with the control (CTL). ^†^ *p* < 0.05 compared with the H_2_O_2_ treatment. ^#^ *p* < 0.05 compared with the ABGE treatment; (**B**) Reduction of the anti-inflammatory effect of FRGE by pretreatment with sugars. Production of NO and PGE_2_ was measured in media collected from LPS-stimulated RAW264.7 cells. * *p* < 0.05 compared with the control (CTL). ^†^ *p* < 0.05 compared with the LPS treatment. ^#^ *p* < 0.05 compared with the FRGE treatment. Each bar represents the mean ± SD of three independent experiments. The plus (+) sign represents conditions with treatment. Pyru, pyruvate; Galac, galactose; Glu, glucose; Fruc, fructose; Suc, sucrose.

The decrease in ROS generation by ABGE was significantly blocked by combination with allicin (*p* < 0.05, Figure 3A). In addition, sugars (glucose, sucrose, fructose, and galactose) did not decrease the H_2_O_2_-induced ROS generation in RAW264.7 cells. As shown in Figure 3A, the effect of NAC used as a positive control for reduction of ROS generation was similar to that of ABGE. These results demonstrate that pyruvate is responsible for the strong anti-oxidant activity of ABGE.

### 2.4. Sugars are Responsible for the Low Anti-Inflammatory Effect of ABGE

The effects of allicin, pyruvate, and sugars on the LPS-stimulated increases in NO and PGE_2_ concentrations were investigated. As shown in Figure 3B, FRGE and allicin significantly inhibited the production of NO and PGE_2_ (*p* < 0.05). Pyruvate also decreased the LPS-induced production of NO and PGE_2_, and this effect did not differ from the FRGE treatment. ABGE also decreased production of NO and PGE_2_, but the effect was small compared to effects of FRGE, allicin, and pyruvate. The anti-inflammatory effect of ABGE was smaller than that of pyruvate, indicating that other ingredients in ABGE might block the anti-inflammatory effect of pyruvate. As shown in Figure 3A, sugars did not suppress H_2_O_2_-induced ROS generation. Additionally, sugar did not decrease the LPS-induced production of NO and PGE_2_. The FRGE-induced decreases in NO and PGE_2_ levels were significantly suppressed in the combined treatment with sugars (*p* < 0.05, Figure 3B), indicating that high concentrations of sugars might be related to inflammatory processes.

### 2.5. Sugars Induce NF-κB Activation

**Figure 4:**
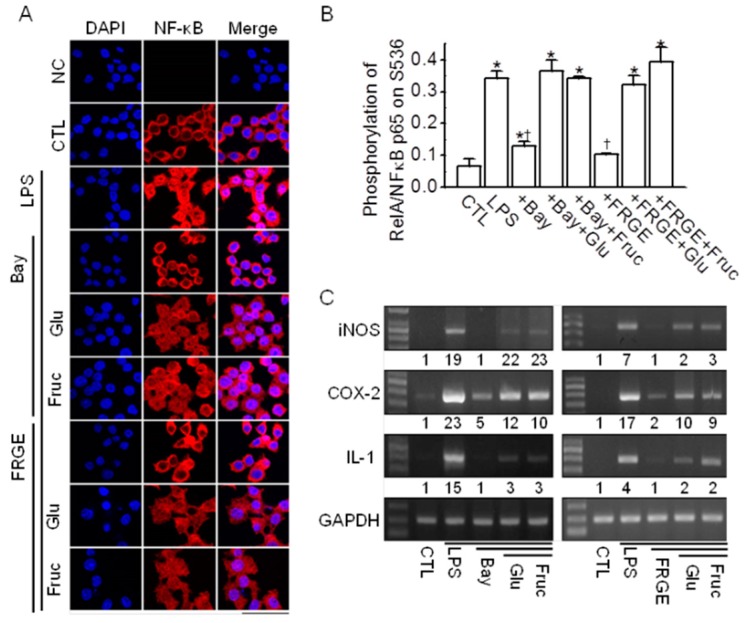
Sugars block the anti-inflammatory effect of FRGE in LPS-stimulated RAW264.7 cells through NF-κB activation. (**A**) Nuclear translocation of NF-κB upon treatment with FRGE and either glucose or fructose. RAW264.7 cells were pretreated with Bay 11-7085, FRGE, and/or glucose or fructose for 12 h and then exposed to LPS for 1 h. Fluorescent cells are labeled with the NF-κB-specific antibody and Cy3-conjugated anti-rabbit IgG. The nucleus was stained with DAPI. The primary antibody was omitted in the negative control (NC). No red fluorescence was observed in the NC; only the DAPI stain was observed (blue color). The scale bar represents 50 μm; (**B**) FRGE-induced NF-κB suppression was decreased by co-treatment with glucose or sucrose in LPS-stimulated RAW264.7 cells. Total and phosphorylated NF-κB p65 levels were analyzed by ELISA using total cell lysate. Each bar represents the mean ± SD of three independent experiments. The plus (+) sign represents conditions with treatment. * *p* < 0.05 compared with the control (CTL). ^†^ *p* < 0.05 compared with the LPS treatment; (**C**) FRGE-induce reduction in the expression of iNOS, COX, and IL-1 mRNA was decreased by treatment with glucose or fructose in LPS-activated RAW264.7 cells. Total cell lysates were obtained using lysis buffer and subjected to RT-PCR. First-strand cDNAs were synthesized from 1 μg of total RNA isolated from the RAW264.7 cells, and the same concentration was used as the template for PCR. GAPDH was used as a loading control for the mRNA expression levels. The numbers below the PCR band represent the normalized ratio of the expression levels of iNOS, COX-2, and IL-1 to those of GAPDH for each lane. Bay, Bay 11-7085; Glu, glucose; Fruc, fructose.

To identify the mechanism by which high concentrations of sugars block the anti-inflammatory effects of garlic extracts, we analyzed NF-κB activation and the expression of pro-inflammatory genes. LPS induced NF-κB activation and exhibited cytoplasm-to-nucleus translocation; this activation decreased with Bay 11-7085, which is an NF-κB inhibitor. However, the effect of Bay 11-7085 decreased upon combining with glucose or fructose. Additionally, FRGE blocked the LPS-induced NF-κB translocation, as Bay 11-7085 did. Co-treatment with glucose or fructose decreased the ability of FREG to block the LPS-induced NF-κB translocation (Figure 4A). The NF-κB activation was validated by measuring phosphorylated NF-κB using fluorogenic substrates. Phosphorylation of p65 at S536 increased with the LPS treatment, but this phosphorylation decreased upon treatment with Bay 11-7085 or FRGE. Co-treatment with glucose or fructose blocked the effects of Bay 11-7085 or FRGE on the LPS-induced phosphorylation of NF-κB p65 (Figure 4B). We determined whether glucose and fructose increase expression of pro-inflammatory genes through NF-κB activation. As shown in Figure 4C, treatment with Bay 11-7085 or FRGE decreased the LPS-induced increase in expression of iNOS, COX-2, and IL-1. Co-treatment with glucose or fructose inhibited the effects of Bay 11-7085 and FRGE, indicating that the inflammatory effects exhibited by glucose and fructose are due to NF-κB activation and transcription.

## 3. Discussion

We hypothesized that ABGE, which has strong anti-oxidant activity, would also have high anti-inflammatory activity because it has been believed that anti-oxidant activity positively correlates with anti-inflammatory effects. In this study, however, ABGE, which has the strongest anti-oxidant effects among garlic extracts, showed low anti-inflammatory activity compared with FRGE. The relationship between anti-oxidant and anti-inflammatory activities of ABGE was not directly proportional. A recent study has reported that ABGE shows anti-inflammatory effects by decreasing the production of NO and pro-inflammatory cytokines with less cytotoxicity in LPS-induced RAW264.7 cells and septicemic mice [6], but the anti-inflammatory activity of ABGE is lower than that of FRGE. These results led us to study compounds that might cause the gap between anti-oxidant and anti-inflammatory effects in ABGE.

The effect of pyruvate was expected, because pyruvate has potent anti-oxidant and anti-inflammatory effects [32,33]. Pyruvate showed strong anti-oxidant and anti-inflammatory activities, which is consistent with earlier studies. However, the anti-oxidant effect of pyruvate was lower than that of ABGE, indicating that other compounds in ABGE likely work with pyruvate to aid in anti-oxidant activities. The concentrations of total phenolics, flavonoids, and thiosulfates increased during the processing of ABGE [16,22]. In addition, organosulfur compounds, such as SAC, DAS, DADS, and DATS, showing anti-oxidant effects are also increased [17,18,34]. These components, along with pyruvate, might act as anti-oxidants in ABGE. Although ABGE has strong anti-oxidant activity, it did not affect NO concentration, which is related with anti-oxidant activity as well as anti-inflammatory activity. In our study, ABGE showed NO radical scavenging activity, but the activity decreased when ABGE was treated to the RAW264.7 cells stimulated by LPS. These differences could result from signaling pathways and factors regulated in cells stimulated by LPS.

The anti-inflammatory effect of pyruvate was markedly high compared with that of ABGE, indicating that other compounds in ABGE might offset the anti-inflammatory effect of pyruvate. Previous studies have shown that ABGE contains a high concentration of sugar (glucose, fructose, galactose, and sucrose) [22,35,36]. Sugars in ABGE produce sweetness and anti-oxidant effects [36]. However, numerous studies have reported that sugars, such as sucrose, fructose, and glucose, enhance inflammation [26,27,37]. In this study, treatment with sugars decreased neither ROS generation in H_2_O_2_-activated RAW264.7 cells nor NO and PGE_2_ production in LPS-activated RAW264.7 cells. In particular, co-treatment of sugars with FRGE reduced the anti-inflammatory activity of FRGE in the generation of NO and PGE_2_. The high sugar concentrations may yield low anti-inflammatory activity from ABGE. The sugar concentration, particularly fructose, in ABGE is approximately 10 times that in FRGE [38]. Excessive sugar concentrations increase pro-inflammatory genes and cytokines through transcription factors, such as NF-κB [39]. A high amount of sucrose in the diet counteracts the anti-inflammatory effect of fish oil in adipose tissue [37]. Glucose and fructose induce increases in plasma concentration of IL-6 and TNF-α, and fructose is more pro-inflammatory than glucose [26,40]. In our study, treatment with glucose or fructose inhibited neither the activation of NF-κB nor the expression of iNOS, COX-2, and IL-1. Treatment with sugars blocked the effects of Bay11-7085 and FRGE on the activation of NF-κB and the expression of iNOS, COX-2, and IL-1, although the concentration was >2000 times lower than real sugar concentration in ABGE.

ABG has been developed as a functional food with anti-oxidant and anti-inflammatory activities. ABG is produced by the Maillard reaction, which caramelizes sugars. Therefore, ABG has a sweet and caramelized taste. ABG features a low concentration of allicin but a high concentration of phytochemicals with anti-oxidant activity compared with FRG. The compound allicin, which produces FRG’s distinctive odor, is converted into SAC, an anti-oxidant compound, during the long heating procedure. SAC and its metabolites, but not allicin, are detected in the plasma, liver, and kidney after oral intake [41,42]. In addition, aged garlic does not damage gastrointestinal mucosa, which is in contrast to FRG [43]. ABG has many health benefits, but it is difficult to conclude that ABG is better for health than is FRG. Heating procedures reduce the anti-inflammatory effects of FRG [8]. If ABG had a strong anti-inflammatory and anti-oxidant effect, ABG would be the best candidate for treating inflammatory diseases induced by oxidative stress. However, the anti-oxidant activity of ABGE is not directly proportional to its anti-inflammatory activity. FRG and ABG should be considered carefully as functional foods because they produce different effects.

## 4. Materials and Methods

### 4.1. Cell Culture and Reagents

The mouse macrophage cell line RAW264.7 was obtained from the American Type Culture Collection (ATCC, Manassas, VA, USA). The cells were cultured in DMEM supplemented with 10% fetal bovine serum (FBS), penicillin (100 U/mL), and streptomycin (100 μg/mL) at 37 °C with 5% CO_2_. The medium was replaced every 2 days. All of the chemicals used in this study were purchased from Sigma (St. Louis, MO, USA) unless otherwise specified.

### 4.2. Preparation of Water-Soluble Garlic Extracts

The FRG was cultivated and harvested in Namhae (Korea). FRGE and HRGE were prepared as described previously [8]. Briefly, crushed FRG was suspended with five volumes of distilled water (DW). Suspended FRG was extracted for 2 h at different temperatures (25 °C and 95 °C for the FRGE and HRGE, respectively) with shaking in a water bath. ABGE was produced from FRG according to the method described in a patent [44]. Briefly, for ABG preparation, FRG was incubated for 48–90 h at 80–90 °C followed by 48–60 h at 70–80 °C, then 72–120 h at 60–70 °C, and finally 72–120 h at 55–65 °C. ABG was suspended with five volumes of DW. The suspended ABG was extracted for 2–6 h at 80–100 °C. The water extract was filtered twice through four pieces of Cheesecloth (Kavon Filter Products Co., Farmingdale, NJ, USA), freeze-dried, powdered, and stored at −20 °C until further analysis. At the time of the experiment, the dried materials were dissolved in DW at the indicated concentrations.

### 4.3. Measurement of ABTS Radical Scavenging Activity

The 2,2-azinobis-(3-ethylbenzothiazoline-6-sulfonate) (ABTS) radical scavenging activity of garlic extract was measured using the method developed by Re *et al.* [45] with some modifications. Briefly, ABTS radical cation (ABTS^+^) was prepared by reacting 7 mM ABTS solution with 2.4 mM potassium persulfate in the dark overnight at room temperature. The ABTS^+^ was adjusted to an absorbance of 1.5 at 415 nm. Equal quantities of garlic extract were mixed with ABTS^+^ and reacted at room temperature for 10 min; the absorbance was then measured at 415 nm using a microplate reader.

### 4.4. Measurement of DPPH Radical Scavenging Activity

The DPPH (2.2-diphenyl-1-picrylhydrazyl) radical scavenging activity of garlic extract was evaluated using a method modified from Blois [26]. Briefly, a freshly prepared DPPH solution (5 mg/100 mL in methanol) was added to each extract. After shaking, the mixture was incubated for 20 min in darkness. Absorbance was then measured at 525 nm using a microplate reader.

### 4.5. Measurement of Cyclooxygenase-2 (COX-2)

The percentage of COX-2 inhibition was measured as described by Copeland RA *et al.* [27] with some modifications. Briefly, the assay mixture contained 450 μL of Tris-HCl buffer (pH 8.0, 100 mM), 100 μL of hematin (150 mM), 100 μL of EDTA (30 μM), 200 μL of COX-2 (40 U/mL), and 100 μL of garlic extract. The mixture was incubated for 15 min at room temperature. The reaction was initiated by adding 20 μL of arachidonic acid (20 mM) and 25 μL of *N,N,N′,N′*-tetramethyl-*p*-phenylenediamine (TMPD, 10 mM) and evaluated after 5 min at 590 nm. EGCG was used as a positive control. The percentage of COX-2 inhibition was calculated using the following equation:

Inhibition (%) = (A_EGCG_ − A_sample_)/A_EGCG_ × 100 (1)

### 4.6. Measurement of 5-Lipoxygenase (5-LO)

The percentage of 5-LO inhibition was measured as described by Lyckander and Malterud [46] with some modifications. Briefly, 200 μL of the enzyme solution (160 U/mL) was prepared in 0.2 M boric acid buffer (pH 9.0), mixed with 50 μL of garlic extract (50, 100, and 250 μg/mL in boric acid buffer), and then incubated at room temperature for 3 min. The reaction was initiated by adding 250 μL of substrate solution (linoleic acid, 100 μM) and evaluated for 2 min at 234 nm. Boric acid buffer and EGCG were used as the negative and positive controls, respectively. The percentage of inhibition of 5-LO was calculated according to the equation used for COX-2.

### 4.7. Measurement of Allicin, Free Sugar, Pyruvate, Thiosulfate, and SAC

The allicin concentration in garlic was measured using the method modified by Miron *et al.*, [47] with some modification. Briefly, each garlic extract powder (10 mg) was dissolved in 10 mL of 50 mM Na-phosphate buffer containing 2 mM EDTA (pH 7.2). The garlic solution (0.1 mL) was mixed with 0.9 mL of 4-mercaptopyridine (10^−4^ M). The absorbance was measured at 324 nm after incubation at room temperature for 30 min. A negative control was obtained using the same procedure without garlic solution. The concentration of allicin was calculated based on the negative control.

The free sugars were determined with HPLC analysis. Briefly, each garlic extract powder (1 g) was dissolved in DW (30 mL) and centrifuged at 4000 rpm (2874 g) for 10 min. The supernatant diluted with DW (50 mL) was filtered through 0.45 μm membrane filter. The filtrate was passed through the Sep-Pak C_18_ cartridges and injected into the HPLC (Agilent 1260 Infinity; Agilent, Santa Clara, CA, USA). Chromatography was performed using YMC-pack Polyamine ІІ column (4.6 × 250 mm; YMC, Kyoto, Japan). The column temperature was 35 °C. The mobile phase was composed of a DW and acetonitrile, and the flow-rate was set at 1.0 mL/min. The peak assignments were based on the retention times of the single compound of glucose and fructose.

The pyruvate concentration of garlic was measured using the method modified by Schwimmer and Guadagni [48]. Briefly, each garlic powder sample (0.5 g) was dissolved in 10% trichloroacetic acid (5 mL). After standing at room temperature for 5 min, the garlic solution was reacted with 1 mM 2,4-dinitrophenylhydrazine in 2 N HCl at 37 °C for 10 min. The absorbance was measured at 420 nm after adding 0.5 mL of 0.6 N NaOH. The pyruvate concentrations were calculated from a standard curve generated from sodium pyruvate.

The thiosulfate concentration of garlic was evaluated using the method modified by Freeman and Mcbreen [49]. Briefly, crushed garlic was suspended in DW (1 g/3 mL). Suspended crushed garlic was extracted and filtered rapidly. The filtrate was added to twice volumes of hexane and extracted after shaking for 2 min. The absorbance of hexane fraction was measured 254 nm.

Crushed garlic (10 g) was extracted in DW (100 mL) for 1 h and centrifuged at 4000 rpm (2874 g) for 10 min. The supernatant (2 mL) was added to the mixture of DW (1): methanol (1): triethylamine (1), and the solution was evaporated. For derivatization, 10 mL mixture solution (phenylisothiocyanate (0.1): DW (2): methanol (5): triethylamine (0.9)) was added to the first mixture (DW: methanol: trimethylamine). The mixture was evaporated and dissolved in 10 mL of 30% acetonitrile and then filtered through 0.45 μm membrane filter. The filtrate was injected into the HPLC (Agilent 1200 series, Agilent Co., Carlsbad, Australia). Chromatography was performed using Watchers 120 ODS-BP (5 μm, 4.6 mm × 250 mm; Watchers, ISU Industry Corp., Seoul, Korea). The mobile phase was composed of acetonitrile and 0.1% acetic acid. Column temperature was 30 °C. The flow-rate was set at 1.0 mL/min. The peak assignments were based on the retention times of the single compound of SAC.

### 4.8. Cell Viability Assay

Cell viability was determined colorimetrically using 3-(4,5-dimethylthiazole-2-yl)-2,5-diphenyl tetrazolium bromide (MTT, Duchefa, Haarlem, The Netherlands). The MTT assay procedures were performed as described previously [50]. Briefly, cells at the exponential phase were seeded in a 24-well plate (2 × 10^4^ cells/well). After 24 h of treatment with an extract or a chemical, 20 µL of 5 mg/mL MTT solution was added to each well (0.1 mg/mL) and incubated for 4 h. The supernatants were then aspirated, the formazan crystals in each well were dissolved in 200 µL of dimethyl sulfoxide (DMSO) for 30 min at 37 °C, and the 24-well plates were read at 570 nm using a microplate reader (Bio-Rad, Hercules, CA, USA). Data are expressed as a percentage of viable cells compared with the control.

### 4.9. Measurement of ROS Generation

The cells were cultured for 3 h with or without 0.1% garlic extract or 3 mM NAC during exposure with 100 μM H_2_O_2_. Briefly, the cells were loaded with 5 μM dichlorodihydrofluorescein diacetate (H_2_DCFDA, Calbiochem, San Diego, CA, USA) for 30 min in darkness. After incubation, the cells were washed three times with phosphate-buffered saline (PBS) and analyzed for fluorescence intensity using a confocal laser scanning microscope equipped with a fluorescence system (IX70 Fluoview, Olympus, Tokyo, Japan). For the detection of green fluorescence (H_2_DCFDA), cells were illuminated with 488- and 519-nm laser lines. The fluorescent images were saved in the tagged image file format and analyzed using Fluoview software (version 2.0, Olympus).

### 4.10. Measurement of Nitrite Levels

Nitrite levels were determined using the Griess method, as described previously [50]. Briefly, RAW264.7 cells (2 × 10^4^/well) were cultured in 96-well plates, incubated overnight, and stimulated with LPS (1 µg/mL) for 24 h. The LPS was obtained from *Escherichia coli* O111:B4. Cells were treated with garlic extract 2 h prior to LPS treatment. Supernatants were then collected, mixed with equal volumes of Griess reagent (1% sulfanilamide, 0.1% *N*-1-naphthylethylendiamine dihydrochloride, and 2% phosphoric acid), and incubated for 10 min. The optical density was measured at 550 nm, and the nitrite levels were calculated from a standard curve generated from sodium nitrite.

### 4.11. Reverse Transcriptase-Polymerase Chain Reaction (RT-PCR)

First-strand cDNAs were synthesized from total RNA isolated from RAW264.7 cells using oligo dT (SuperScript™ First-Strand Synthesis System; Invitrogen, Carlsbad, CA, USA). The first-strand cDNA was used as a template for PCR amplification. Specific primers for mouse iNOS, COX-2, and IL-1α were used with Taq polymerase (G-Taq, Cosmo Genetech, Seoul, Korea). Details of primer sequences are shown in Table 2. The PCR conditions for RT-PCR were as follows: initial denaturation at 94 °C for 5 min; 27–30 cycles at 94 °C for 30 s, 55 °C for 30 s, and 72 °C for 30 s; and a final extension step at 72 °C for 10 min. The products were electrophoresed on 1.5% (*w*/*v*) agarose gels to verify the product size. The DNA fragments were directly sequenced with the ABI PRISM^®^ 3100-Avant Genetic Analyzer (Applied Biosystems, Carlsbad, CA, USA).

### 4.12. Western Blot Analysis

Proteins isolated from the RAW264.7 cells were prepared as total protein. Western blot analysis was performed by lysing the cells in RIPA buffer (25 mM Tris-HCl; pH7.4, 150 mM NaCl, 1% NP-40, 1% sodium deoxycholate, and 0.1% SDS) containing protease inhibitors. The procedures were as described previously [50]. The membranes were blocked with 5% fat-free dry milk and then incubated with an anti-COX-2 (1:1000 dilution, Cell Signaling Technology, Beverly, MA, USA) or anti-β-actin antibody (1:10,000 dilution). Next, the membranes were incubated with a peroxidase-conjugated anti-rabbit or anti-mouse secondary antibody at 1:10,000 (Assay Designs, Ann Arbor, MI, USA). Immuno-positive bands were visualized with an enhanced chemiluminescence reagent (Dogen, Seoul, Korea) according to the manufacturer’s instructions.

### 4.13. Immunocytochemistry

RAW264.7 cells were cultured on round cover slips coated with poly-L-lysine, pretreated with chemicals and/or FRGE for 12 h, and then stimulated with LPS for 1 h. After washing with PBS (pH 7.4), the cells were fixed with 4% paraformaldehyde for 30 min. The cells were washed again with PBS, permeabilized with 0.2% Triton X-100 for 20 min at room temperature, and incubated with an anti-NF-κB p65 antibody (1:100 dilution, Cell Signaling Technology) at 4 °C overnight. After three washes in PBS, the cells were incubated in the dark for 1 h with Cy3-conjugated anti-rabbit IgG fluorescent secondary antibody diluted 1:500 in PBS. Finally, the cells were washed and stained with 4′,6′-diamidino-2-phenylindole (DAPI) for nuclei staining. The stained cells were wet-mounted on glass slides and observed using a confocal laser scanning microscope (Olympus). The negative controls (NCs) were analyzed by omitting the primary antibody.

### 4.14. Enzyme-Linked Immunosorbent Assays (ELISA) for Pro-Inflammatory Cytokines and NF-κB

For the immunoassays, cells (1 × 10^6^ cells/mL) were cultured in 6-well plates, incubated overnight, and stimulated with LPS (1 μg/mL) for 24 h. The cell supernatant was then collected, and the levels of IL-1β, IL-6 (KOMA Bio-tech., Seoul, Korea), prostaglandin E_2_ (PGE_2_) (R & D Systems, Minneapolis, MN, USA), and leukotrienes (LTD4 and LTE4) (MyBioSource, San Diego, CA, USA) were quantified using ELISA kits according to the manufacturer’s instructions.

NF-κB activation in RAW264.7 cells was analyzed using a Phospho-RelA/NF-κB p65 (S536) immunoassay kit (R & D Systems) according to the manufacturer’s instructions. Briefly, RAW264.7 cells (1 × 10^4^ cells/well) were cultured in a black 96-well microplate with clear bottoms and incubated overnight. The cells were pretreated with chemicals and/or FRGE for 3 h and stimulated with LPS for 8 h. Then, the cells were fixed, permeabilized, and blocked. The cells were incubated with 100 μL of a primary antibody mixture at 4 °C overnight. After washing three times with washing buffer, the cells were incubated for 2 h with 100 μL of secondary antibody mixture at room temperature and then washed three times. The cells were incubated in the dark with 75 μL of substrate F1 for 20 min and 75 μL of substrate F2 for 30 min. Fluorescence of the plates was determined at 540 nm excitation and 600 nm emission and then at 360 nm excitation and 450 nm emission using a fluorescence microplate reader (Tecan, Maennendorf, Switzerland). The 600 nm data indicate the quantity of phosphorylated RelA/NF-κB p65 in the cells, while the 450 nm data indicate the quantity of total RelA/NF-κB p65 in the cells. The normalized results were determined by dividing the phospho-RelA/NF-κB p65 fluorescence by the total RelA/NF-κB p65 fluorescence in each well.

### 4.15. Data Analysis and Statistical Analysis

An LAS-4000 (Fujifilm Corp, Tokyo, Japan) luminescent image analyzer was used to capture images of western blots. The bands were quantified using ImageJ software (version 1.49, National Institute of Health, Bethesda, MD, USA). The relative protein levels were calculated using β-actin as a loading control. One-way ANOVA (SPSS 18, Chicago, IL, USA) with post hoc comparisons using Tukey’s test was used with *p* < 0.05 as the criterion for significance. The data are presented as the mean ± standard deviation.

## 5. Conclusions

In conclusion, the lower anti-inflammatory activity of ABGE compared with FRGE may result from sugars, such as galactose, glucose, fructose, and sucrose. Our results suggest that ABGE might aid in treating predominantly ROS-mediated diseases.

## Figures and Tables

**Table 1:** Chemical components in garlic extracts.

Type of Garlic Extracts	Pyruvate (μM/g)	Thiosulfinate (μM/g)	Free Sugars (mg/g)		Allicin (mg/g)	SAC (mg/g)
			Glucose	Fructose		
**FRGE**	486.71 ± 12.08	6.50 ± 0.29	**-**	7.07 ± 0.08	3.62 ± 0.01	1.24 ± 9.22
**HRGE**	1023.90 ± 9.83	6.61 ± 0.25	0.21 ± 0.02	7.30 ± 0.25	1.93 ± 0.02	1.82 ± 8.24
**ABGE**	2456.54 ± 23.93	91.22 ± 0.54	2.10 ± 0.05	40.02 ± 0.71	**-**	2.12 ± 10.17

**Table 2:** Primer sequences used for RT-PCR.

Gene Name	GenBank Acc. No.	Primer Sequences (5′–3′)	Expected Size (bp)
iNOS	NM_010927	Sense: TTGACGCTCGGAACTGTAGCA	492
		Antisense: TGCCCATGTACCAACCATTGA	
COX-2	NM_011198	Sense: CCCAGAGCTCCTTTTCAACCA	382
		Antisense: TGCAGCCATTTCCTTCTCTCC	
IL-1	NM_010554	Sense: GGAGAGCCGGGTGACAGTAT	364
		Antisense: GGGCTGGTCTTCTCCTTGAG	
IL-6	NM_031168	Sense: CTTCACAAGTCCGGAGAGGAG	489
		Antisense: TGGTCTTGGTCCTTAGCCACT	
GAPDH	GU214026	Sense: CTA AAG GGC ATC CTG GGC	201
		Antisense: TTA CTC CTT GGA GGC CAT	

## Abbreviations

The following abbreviations are used in this manuscript:

ABGE: Aged black garlic extract
EGCG: Epigallocatechin gallate
FRGE: Fresh raw garlic extracts
HRGE: Heated raw garlic extract
NAC: N-acetyl-L-cysteine
